# Investigation of Solidification Heat Transfer in Slab Continuous Casting Process Based on Different Roll Contact Calculation Methods

**DOI:** 10.3390/ma17020482

**Published:** 2024-01-19

**Authors:** Daiwei Liu, Guifang Zhang, Jianhua Zeng, Chenhui Wu

**Affiliations:** 1 Faculty of Metallurgical and Energy Engineering, Kunming University of Science and Technology, Kunming 650093, China; liudaiwei158@163.com; 2 School of Electrical and Information Engineering, Panzhihua University, Panzhihua 617000, China; 3 Pangang Group Research Institute Co., Ltd., Panzhihua 617000, China; zengjianhua68@163.com (J.Z.); wch_neu@126.com (C.W.)

**Keywords:** wide-thick slab, heat transfer, uneven cooling, roll contact

## Abstract

The heat transfer of a slab is significantly influenced by roll contact during the continuous casting process. The influence of roll contact calculation methods on the predicted heat transfer results has not been previously investigated. In this work, the non-uniform solidification of the wide-thick slab was studied with a 2D heat transfer model using real roll contact method (R. method) and equivalent roll contact method (E. method). The predicted slab surface temperature and shell thickness were verified with the measured results of the infrared camera and nail shooting experiments, respectively. Then, the predicted heat transfer results (including the slab surface temperature, mushy region length, and solidification end position) for the wide-thick slab with different thicknesses and different casting speeds were calculated using the E. method and R. method, and the influence of these two roll contact methods on the predicted heat transfer results was discussed for the first time. The results show that both these two roll contact methods could be applied to accurately predict the slab surface temperature without considering the transient temperature dips in the roll–slab contact regions. However, the deviation of the predicted mushy region length and solidification end position using the E. method are obvious. Compared with the R. method, the predicted mushy region length obtained using the E. method is larger and the solidification end obviously subsequently moves along the casting direction.

## 1. Introduction

In the continuous casting process, the heat of the liquid steel is sequentially removed in the mold, the secondary cooling region, and the air cooling region, which is closely related to the quality and productivity of the casting steel [1,2,3]. Many researchers [4,5,6,7,8,9,10,11,12,13] have studied the heat transfer behavior of casting steel using numerical calculation methods in order to optimize the cooling and other process parameters. Based on the predicted slab surface temperature and the target temperature, the cooling water amount or nozzle arrangement in the secondary cooling region was optimized to eliminate the slab surface defects of transverse cracks [4,5,6,7]. Furthermore, according to the predicted features of the mushy region and the strand position of the solidification end, a reasonable position for implementing strand/final electromagnetic stirring (S/F-EMS) [8,9,10] or soft/heavy reduction (S/HR) [11,12,13] was determined to effectively improve the internal defects of centerline segregation and porosity.

**Figure 1:**
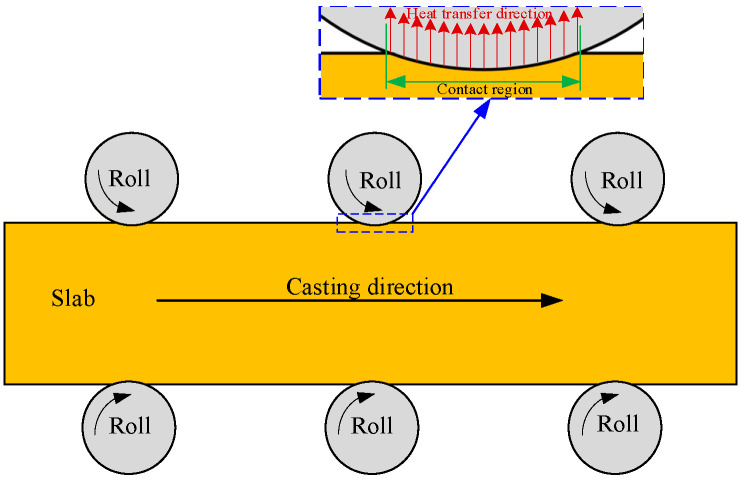
Schematic of heat transfer in roll–slab contact region.

Roll contact (shown in Figure 1), as one of the main cooling methods after the mold, removes about 10% [14] of the heat of the casting steel and thus significantly influences the heat transfer of the continuously cast steel. In the previous works, two main calculation methods were adopted to deal with the cooling boundary condition between rolls and strand in the numerical study of the strand heat transfer behavior: the equivalent roll contact method (E. method) and the real roll contact method (R. method). For the E. method [11,15,16], all the heat extracted via roll contact is regarded as a uniformly releasing process along the casting direction, and for the R. method [14,17,18], the cooling boundary condition for roll contact is applied in each roll–slab contact region. For the numerical calculation process of heat transfer using the R. method, the calculation time step should be strictly limited to ensure that all the roll–slab contacts can be detected in order to accurately apply the corresponding cooling boundary condition in the contact regions. If the calculation time step is too large, some roll–slab contact regions cannot be detected, and the cooling effect of some detected roll contacts on the slab heat transfer will be excessively enlarged. Furthermore, due to the high cooing intensity in the roll–slab contact regions (the heat transfer coefficient in the roll–slab contact regions approximately reaches 1000~1300 w/(m^2^·°C) [14]), finer grids should be applied to discretize the calculation domain of the mathematical heat transfer model in order to more accurately consider the cooling effect of the rolls. For the reasons outlined above, the R. method is more complicated, and obviously, the corresponding computational cost consequently increases, whereas because the cooling effect of the roll contact can be considered more practically using the R. method, the accuracy of the predicted heat transfer results obtained using this method are undoubtedly better than those obtained using the E. method.

The present work focuses on the numerical calculation of the wide-thick slab heat transfer under uneven cooling conditions in the continuous casting process and mainly studies the influence of calculation method for roll contact on the predicted slab surface temperature, strand position of the solidification end, and the mushy region length, aiming to provide a theoretical basis for choosing an appropriate roll contact method to accurately and rapidly calculate the relevant heat transfer results during the optimization of some process parameters.

## 2. Heat Transfer Model

### 2.1. Model Description

**Figure 2:**
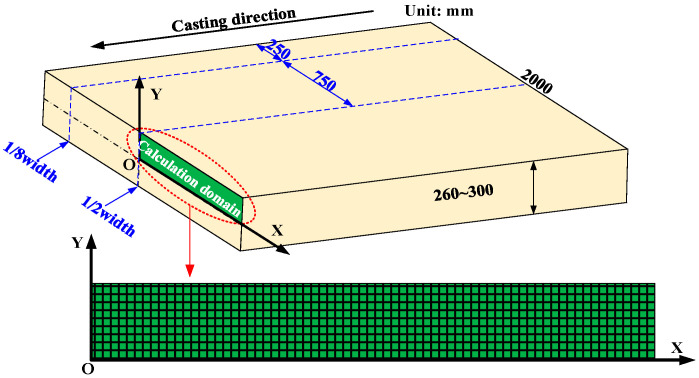
Schematic of the 2D heat transfer model.

In the present work, the peritectic steel slab produced by a commercial wide-thick continuous caster was taken as the specific research object. Based on some simplified assumptions [19], one quarter of the slab transverse section was chosen as the calculation domain to establish the 2D heat transfer model. Four-node rectangular elements with a mesh size of 2 mm × 2 mm were used to discrete the calculation domain, and the final finite element model is shown in Figure 2. During the calculation process, the time step is 0.2 s, and Table 1 lists more detailed parameters about the casting process and the continuous cast.

The heat transfer behavior of the mathematical model can be described by the two-dimensional transient heat conduction equation:

(1) $$\rho C\frac{\partial T}{\partial t}=\frac{\partial }{\partial x}\left(\lambda \frac{\partial T}{\partial x}\right)+\frac{\partial }{\partial y}\left(\lambda \frac{\partial T}{\partial y}\right)$$

where *T* and *t* are, respectively, the temperature in °C and calculation time in s; *ρ*, *c*, and *λ* are the temperature-dependent density, specific heat, and conductivity in kg/m^3^, J/(kg·°C), and w/(m·°C), respectively.

**Figure 3:**
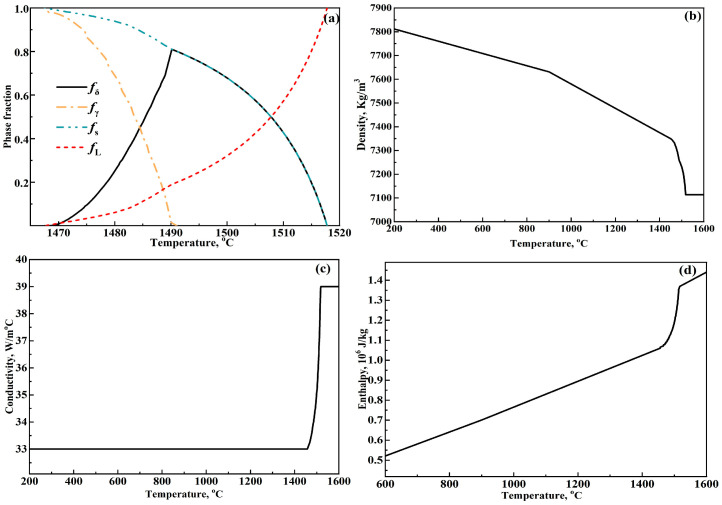
Phase fraction and thermal material properties of peritectic steel: (**a**) phase fraction, (**b**) density, (**c**) conductivity, and (**d**) enthalpy.

To accurately acquire the thermal material properties of the peritectic steel, a microsegregation model that has been described in detail in a previous work [11] was employed to calculate the phase fraction evolution, and the phase fraction evolution is shown in Figure 3a. *f_δ_*, *f_γ_*, *f_s_*, and *f_L_* in Figure 3a respectively represent the fraction of *δ*-Fe, *γ*-Fe, solid, and liquid, and *f_s_* is equal to the sum of *f_δ_* and *f_γ_*. With the temperature decrease, the amount of liquid phase continuously decreases from the liquidus temperature of 1517.7 °C; meanwhile, the amount of solid phase (including *δ*-Fe and *γ*-Fe) continuously increases. During this process, *δ*-Fe precipitate first and *γ*-Fe forms subsequently. When the temperature decreases to the solidus temperature of 1467.5, liquid steel disappears, and the solidification process finishes.

Based on the phase evolution in Figure 3a, the thermal material properties of the research steel, including density, conductivity, and enthalpy, were calculated using weighted phase fraction equations [20] and are respectively shown in Figure 3b, Figure 3c, and Figure 3d. With the temperature increase, density in Figure 3b continuously decreases, while conductivity in Figure 3c and enthalpy in Figure 3d present an overall increasing trend. It should be noted that the thermal conductivity of the liquid steel is magnified by 1.5 times compared to that in the solid state when considering the improving effect of molten steel flow on heat conduction of steel [4,11].

### 2.2. Boundary Conditions

The casting temperature, 1548 °C, is taken as the initial temperature of the heat transfer model, and heat flux at symmetrical sides (OX, OY) is set as zero. During the calculation process, the 2D heat transfer model was assumed to move with casting speed from meniscus to the continuous caster end. Corresponding cooling boundary conditions are applied according to the strand position of the 2D heat transfer model, and the calculation methods for cooling boundary conditions in mold and out of mold are described as follows:

(1) In mold:

The heat flux between the strand surface and mold can be calculated with the following equation proposed by Savage and Pritchard [21]:

(2) $$q=A-B\sqrt{t}$$

where *q* is heat flux between the solidified shell and mold, Mw/m^2^; *A* and *B* are coefficients depending on the mold cooling condition; *t* is calculation time in mold, s.

(2) Out of mold:

When the slab moves out of mold, heat of the strand is mainly taken away by sprayed cooling water, roll contact, and radiation.

**Figure 4:**
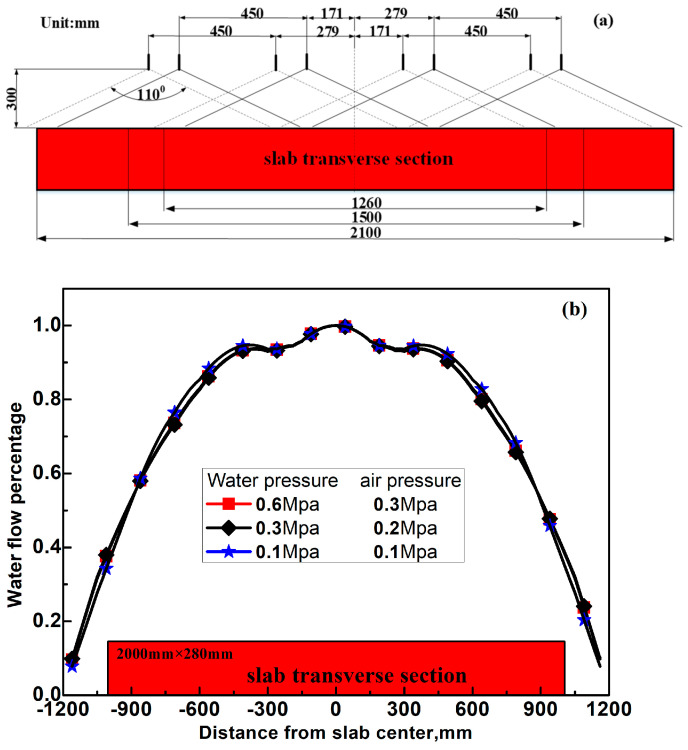
(**a**) Nozzle arrangement and (**b**) the corresponding measured water flux distribution along the slab width in zones 5–8.

(i) For sprayed cooling water [19]:
(3) $$h_{spray}^{i}=\alpha_{i}\;\cdot \;{W_{i}}^{0.55}\left(1-0.075T_{w}\right)$$
  where *i* denotes the *i*th secondary cooling zone; $h_{spray}^{i}$ is the heat transfer coefficient between the strand surface and cooling water, w/(m^2^·°C); *T_w_* is the cooling water temperature, °C; *W_i_* represents the cooling water flux density, L/(m^2^·min). As the water flux distribution along the slab width direction in secondary cooling Zone 5~Zone 8 is obviously non-uniform, the water flux distribution in these secondary cooling zones was measured and applied in the calculation of $h_{spray}^{i}$ . Figure 4a and Figure 4b show the nozzle arrangement and the corresponding measured water flux distribution, respectively.
(ii) For roll contact [16,17] with the E. method:
(4) $${\bar{h}}_{con}^{i}=\frac{h_{R/s}^{i}\;\cdot \;N_{R}^{i}\;\cdot \;L_{R/S}}{L^{i}}$$
  where *i* denotes the *i*th secondary cooling zone; ${\bar{h}}_{con}^{i}$ is the equivalent heat transfer coefficient between the strand surface and rolls, w/(m^2^·°C); $N_{R}^{i}$ is the number of rolls; $h_{R/s}^{i}$ denotes the real heat transfer coefficient between the slab and rolls, w/(m^2^·°C); *L_R/S_* is the length of each roll–slab contact region, m. According to a previous work [14], $h_{R/s}^{i}$ ranges from 1000 w/(m^2^·°C) to 1300 w/(m^2^·°C), and *L_R/S_* was set as 0.02 m; *L^i^* represents the length of the *i*th cooling zone, m.
(iii) For radiation:
(5) $$h_{rad}=\varepsilon \;\cdot \;\sigma \;\cdot \;\left(T_{surf}^{2}+T_{env}^{2}\right)\;\cdot \;\left(T_{surf}+T_{env}\right)$$
  where *h_rad_* is the heat transfer coefficient of radiation, w/(m^2^·K); *ε* is the emissivity, 0.8 [16]; *σ* is the Stefan–Boltzmann coefficients, 5.67 × 10^−8^(w/m^2^ K^4^); *T_surf_* and *T_env_* denote the temperature of strand surface and the environment, respectively, K.

Based on the abovementioned calculation formulas for the heat transfer coefficients of sprayed cooling water, roll contact, and radiation, the cooling boundary conditions out of mold can be expressed with two forms of equations according to the adopted calculation method for roll contact:

E. method:

(6) $$-\lambda \frac{\partial T}{\partial n}=\left(h_{spray}^{i}+{\bar{h}}_{con}^{i}+h_{rad}\right)\;\cdot \;\left(T_{surf}-T_{env}\right)$$

R. method:

(7) $$-\lambda \frac{\partial T}{\partial n}=\left(1-k\right)\;\cdot \;\left(h_{spray}^{i}+h_{rad}\right)\;\cdot \;\left(T_{surf}-T_{env}\right)+k\;\cdot \;h_{R/s}\;\cdot \;\left(T_{surf}-T_{Roll}\right)$$

where *T_Roll_* is the roll surface temperature and set as 150 °C according to the previous work of Xia [14]; *k* denotes whether the 2D heat transfer model is in the roll–slab contact regions: if in, then *k* = 1, but if not in, then *k* = 0. Additionally, because the cooling water sprayed on the strand surface disappears in the air cooling zone, $h_{spray}^{i}$ is equal to zero in this cooling region.

### 2.3. Model Validation

**Figure 5:**
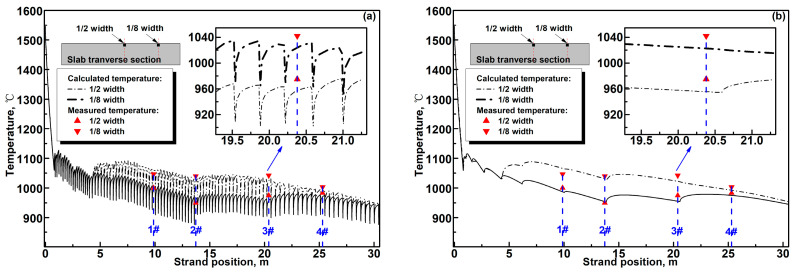

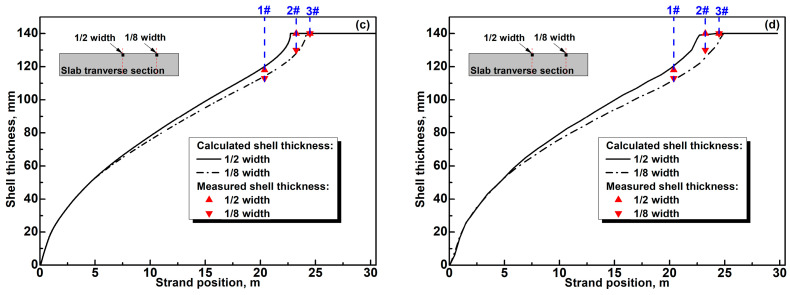
Comparison of the calculated and measured results: (**a**) calculated temperature using the R. method, (**b**) calculated temperature using the E. method, (**c**) calculated shell thickness using the R. method, and (**d**) calculated shell thickness using the E. method.

To evaluate the calculation accuracy of the R. method and E. method, the slab surface temperature and shell thickness were measured via infrared camera and nail shooting experiments at different locations in the transverse direction and casting direction; the 2000 mm × 280 mm slab was cast at 0.8 m/min. Table 2 shows the specific parameters about the measuring positions, and the measured results are compared with the calculated ones using the R. method and E. method in Figure 5a–d.

In Figure 5, the temperature and shell thickness at 1/8 width of the wide-thick slab are, respectively, higher and thinner than that at 1/2 width due to the continuously declining cooling water flux from the slab surface center to corner as shown in Figure 4b. In Figure 5a, transient slab surface temperature dips can be observed in roll–slab contact regions because the cooling effect of the slab surface is dramatically enhanced in these regions.

It can be seen from Figure 5a–c that the predicted slab surface temperature using the R. method and E. method and the predicted shell thickness obtained using the R. method agrees well with the measured ones. The maximum absolute value of relative error between the measured slab surface temperature and the predicted ones are less than 1.9% for the R. method and less than 2.6% for the E. method. The maximum absolute value of relative error between the measured shell thickness and the predicted ones obtained using the R. method is less than 1.7%. However, the relative deviation between the measured shell thickness and the predicted ones obtained using the E. method is relatively obvious, and the maximum absolute value of relative error of the predicted shell thickness obtained using the E. method reaches 3.2%.

## 3. Results and Discussion

### 3.1. Difference between the R. Method and E. Method on the Predicted Heat Transfer Results

**Figure 6:**
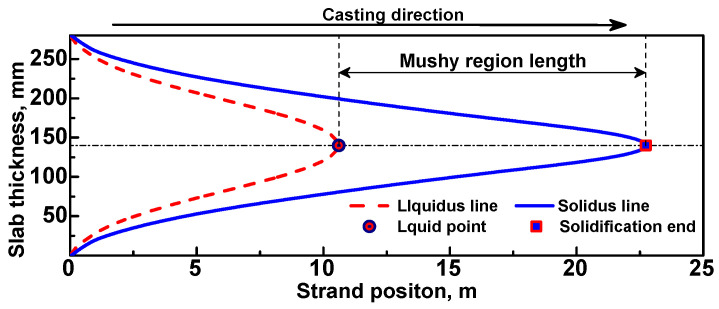
Illustration of the mushy region length and solidification end at the mid-width (1/2 width) plane of a 2000 mm × 280 mm slab produced under a casting speed of 0.8 m/min.

As the slab surface temperature are closely related to the optimization of secondary cooling process [4,5,6,7] and the mushy region and solidification end are an important theoretical basis for determining reasonable process parameters for S/F-EMS [8,9,10] and S/HR [11,12,13], slab surface temperature, mushy region length, and strand position of solidification end are calculated, respectively, using the R. method and E. method in order to study the difference between these two roll contact methods. Figure 6 illustrates the solidification end and mushy region length.

**Figure 7:**
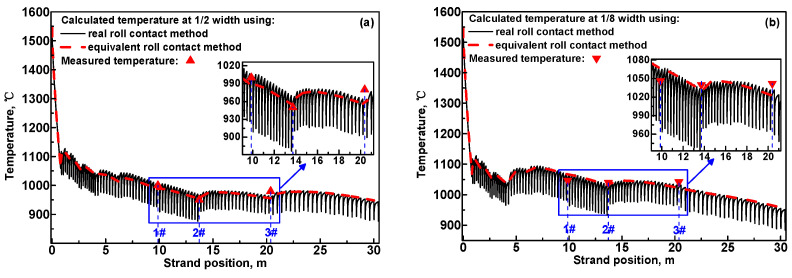
Comparison of the predicted slab surface temperature obtained using different roll contact methods with the measured results at (**a**) 1/2 width and (**b**) 1/8 width.

Figure 7a and Figure 7b, respectively, compare the calculated surface temperature at 1/2 and 1/8 width of the slab using two different calculation methods for roll contact. Compared with the R. method, the transient surface temperature dip in each roll–slab contact region disappears when the calculation was carried out using the E. method. This is because the heat extracted via roll–slab contact was regarded as a uniformly releasing process along the casting direction for the E. method. However, without considering the transient temperature dip in each roll–slab contact region, the predicted slab surface temperature obtained using the E. method is essentially consistent with the predicted results obtained using the R. method, and agree well with the measured results. Therefore, these two calculation methods for roll contact are both applicable for accurately predicting the slab surface temperature during the optimization of secondary cooling process, but the E. method is preferable due to its simplicity and high efficiency.

**Figure 8:**
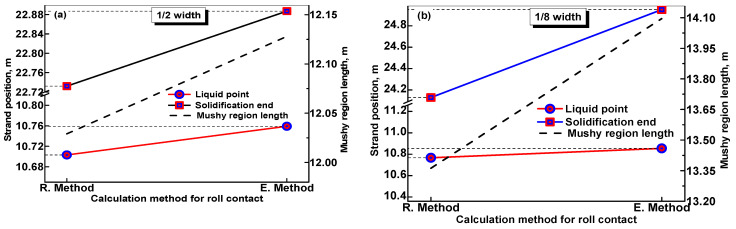
Comparison of the calculated strand position of solidification end and mushy region length using the R. method and E. method at (**a**) 1/2 width and (**b**) 1/8 width plane of a 2000 mm × 280 mm slab produced under the casting speed of 0.8 m/min.

Figure 8a and Figure 8b, respectively, compare the calculated solidification end position and mushy region length using the R. method and E. method at 1/2 width and 1/8 width plane of the wide-thick slab. It is obvious that the predicted strand positions of the solidification end obtained using the E. method shift along the casting direction compared with that predicted using the R. method, which is caused by the neglect of the transient temperature dips in the roll–slab contact regions when the E. method is employed. Figure 7b shows that the calculated solidification end position obtained using the E. method at 1/8 width plane of the slab is 24.95 m. However, the measured shell thickness in Figure 5c,d indicates that the solidification end at 1/8 width plane of the slab is before the 3# measuring position of 24.50 m, which is obviously inconsistent with the predicted result of 24.95 m obtained using the E. method. This proves that the predicted solidification end obtained using the E. method obviously lags behind the real solidification end.

Although both the predicted liquid point and solidification end in Figure 8a,b obtained using the E. method shift along the casting direction compared with that predicted using the R. method, the solidification end shifts more obviously. As a result, the predicted mushy region using the E. method is longer than that predicted using the R. method. Furthermore, the deviation of the predicted solidification end and mushy region length obtained using the E. method are more obvious at 1/8 width plane of the wide-thick slab compared with that at 1/2 width. This is mainly caused by two factors: (1) the higher surface temperature at 1/8 width increases the temperature difference between the slab surface and rolls surface and thus enhances the cooling effect of the rolls; (2) as indicated in Figure 5c,d, the solidification end at 1/8 width is located behind that at 1/2 width due to the continuously declining cooling intensity from the slab surface center to the corner. This leads to the fact that more roll–slab contact regions exist before the solidification end at 1/8 width, which further increases the total heat amount extracted via roll–slab contact.

As the deviation of the predicted solidification end position and mushy region length obtained using the E. method, especially the predicted results at 1/8 width plane of the width-thick slab, are obvious, it is preferable to adopt the R. method to predict the solidification end position and the features of the mushy region, which could provide more reliable data for determining a reasonable location to implement S/HR or S/F-EMS.

### 3.2. Effect of Casting Speeds on the Deviation of the Predicted Results Using the E. Method and R. Method

As the deviation of the predicted solidification end position and the mushy region length obtained using the E. method and R. method are especially obvious at 1/8 width plane of the wide-thick slab, these two results were calculated and compared using the E. method and R. method, respectively, under different casting speeds in order to investigate the influence of casting speeds on the deviation degree of the predicted results obtained using the E. method.

**Figure 9:**
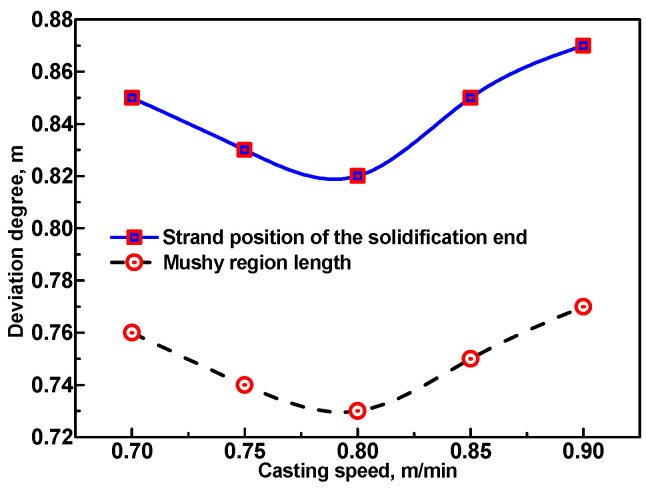
Deviation degree of the predicted strand position of solidification end and mushy region length using the E. method and R. method at 1/8 width plane of the 2000 mm × 280 mm slab under different casting speeds.

Figure 9 shows the deviation degree (the deviation degree is defined as that the predicted strand position of solidification end or mushy region length obtained using the E. method minus the predicted ones obtained using the R. method) of the predicted results obtained using the E. method and R. method under different casting speeds. It can be seen that the deviation degree of solidification end position are overall larger than the deviation degree of mushy region length, and the variation trend of the deviation degree of these two predicted results obtained using the E. method are similar. With the increase in casting speed, two main factors significantly influence the tendency of the deviation degree: (a) the increase in casting speed dramatically shortens the contact time in each roll–slab contact region, which helps to decrease the deviation degree of the predicted results obtained using the E. method which is caused by neglect of the transient temperature dips in roll–slab contact regions. (b) However, with the increase in casting speed, the solidification end subsequently moves along the casting direction, and thus the mushy region length simultaneously increases. This means more roll–slab contact regions exist before the strand solidification end, which tend to enlarge the deviation degree of the predicted results obtained using the E. method. In Figure 9, with the increase in casting speed, the deviation degree first shows a decreasing tendency between 0.7 m/min and 0.8 m/min and then continuously increases in the range of 0.8~0.9 m/min. This indicates that the above-mentioned Factor (a) plays a more significant role in influencing the tendency of the deviation degree when the casting speed is in the range of 0.7–0.8 m/min. However, Factor (b) influences the variation of the deviation degree more obviously when the casting speed is in the range of 0.8–0.9 m/min.

### 3.3. Effect of Slab Thickness on the Deviation of the Predicted Results Using the E. Method and R. Method

**Figure 10:**
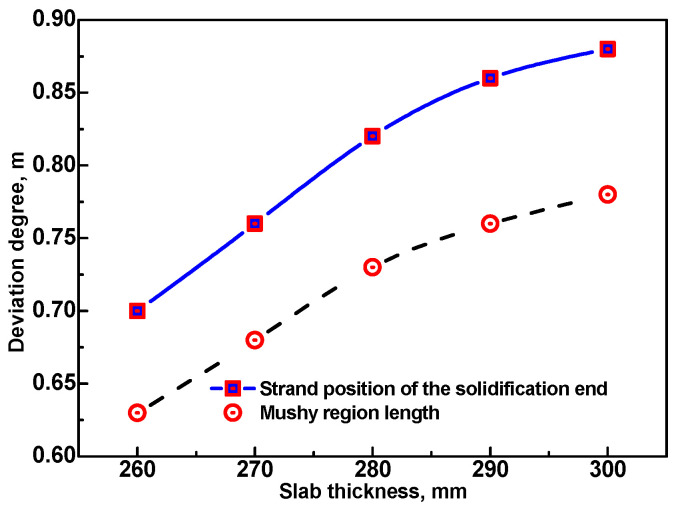
Deviation degree of the predicted solidification end position and mushy region length using the E. method and R. method at 1/8 width plane of the wide-thick slab with different thickness produced at 0.8 m/min.

In order to study the influence of slab thickness on the deviation degree of the predicted strand position of solidification end and mushy region length obtained using the E. method and R. method, these two results at 1/8 width plane of the wide-thick slab with different thickness were calculated and compared using the E. method and R. method, respectively. Figure 10 shows the variation of deviation degree with slab thickness. With the increase in slab thickness, the solidification end subsequently moves along the casting direction, and the mushy region length is also increased. This results in more roll–slab contact regions before the strand solidification end and thus tends to enlarge the deviation degree of the predicted results obtained using the E. method and R. method. However, the increase in slab thickness means more heat per unit length of strand to be extracted during the solidification process due to the amount of secondary cooling water increasing correspondingly, which results in a tendency to decrease the ratio of the heat extracted via roll–slab contact. As a result, the increase in slab thickness also has an effect of helping to decrease the deviation degree of the predicted results obtained using the E. method and R. method. Affected more significantly by the former of the two factors mentioned above, both the deviation degree of the predicted solidification end position and mushy region length obtained using the E. method and R. method continuously increase with the increasing slab thickness. However, the increase in the deviation degree continuously grows slower.

## 4. Conclusions

(1) Both the R. method and the E. method can be used to accurately predict the wide-thick slab surface temperature without considering the transient temperature dips in the roll–slab contact regions, and the E. method is preferable during the optimization of the secondary cooling process due to its simplicity and high efficiency;
(2) With the casting speed increased from 0.7 m/min to 0.9 m/min for the 280 mm thick slab, the deviation of the predicted results obtained at 1/8 width using the E. method ranges from 0.82 m to 0.87 m for the solidification end position and ranges from 0.73 m to 0.77 m for the mushy region length. With the slab thickness increased from 260 mm to 300 mm at the casting speed of 0.8 m/min, the deviation increases from 0.70 m to 0.88 m for the solidification end position and increases from 0.63 m to 0.78 m for the mushy region length;
(3) Deviation of the predicted solidification end position and mushy region length obtained using the E. method is obvious. It is preferable to adopt the R. method to predict the solidification end position and mushy region length in order to provide more reliable data to determine a reasonable location for implementing S/HR or S/F-EMS.

In order to predict the solidification end position and mushy region length more accurately and efficiently using the E. method, a data rectification algorithm for the E. method on predicting solidification end position and mushy region length will be investigated in our future work based on the present work.

## Figures and Tables

**Table 1:** Parameters about the casting process and the continuous caster.

Items	Unit	Value
Steel composition	wt%	C: 0.17 Si: 0.15 Mn: 0.6 P: 0.015 S: 0.01
Liquidus temperature	°C	1517.7
Solidus temperature	°C	1467.5
Slab width	mm	2000
Slab thickness	mm	260~300
Casting speed	m/min	0.7~0.9
Casting temperature	°C	1548
Effective length of mold	mm	800
Each secondary cooling zone length	mm	Zone 1: 240; Zone 2: 560; Zone 3: 1110; Zone 4: 1550; Zone 5: 1920; Zone 6: 3840; Zone 7: 3840; Zone 8: 6725
Air cooling zone length	mm	9840

**Table 2:** Strand positions for temperature measuring.

Measuring No.	Strand Position for Temperature Measuring, m	Strand Position for Shell Thickness Measuring, m
1#	9.87	20.38
2#	13.72	23.25
3#	20.38	24.50
4#	25.30	/

1#−4# means the number of measuring position.

## Data Availability

Data are contained within the article.
